# Genetic control of encoding strategy in a food-sensing neural circuit

**DOI:** 10.7554/eLife.24040

**Published:** 2017-02-07

**Authors:** Giovanni Diana, Dhaval S Patel, Eugeni V Entchev, Mei Zhan, Hang Lu, QueeLim Ch'ng

**Affiliations:** 1 Centre for Developmental Neurobiology, King's College London, London, United Kingdom; 2 Interdisciplinary Bioengineering Graduate Program, Georgia Institute of Technology, Atlanta, United States; 3 Wallace H Coulter Department of Biomedical Engineering, Georgia Institute of Technology, Atlanta, United States; 4 School of Chemical and Biomolecular Engineering, Georgia Institute of Technology, Atlanta, United States; Howard Hughes Medical Institute, Columbia University, United States

**Keywords:** Information Theory, gene expression, neural code, Transforming Growth Factor Beta, Serotonin, *C. elegans*

## Abstract

Neuroendocrine circuits encode environmental information via changes in gene expression and other biochemical activities to regulate physiological responses. Previously, we showed that *daf-7* TGFβ and *tph-1* tryptophan hydroxylase expression in specific neurons encode food abundance to modulate lifespan in *Caenorhabditis elegans*, and uncovered cross- and self-regulation among these genes (Entchev et al., 2015). Here, we now extend these findings by showing that these interactions between *daf-7* and *tph-1* regulate redundancy and synergy among neurons in food encoding through coordinated control of circuit-level signal and noise properties. Our analysis further shows that *daf-7* and *tph-1* contribute to most of the food-responsiveness in the modulation of lifespan. We applied a computational model to capture the general coding features of this system. This model agrees with our previous genetic analysis and highlights the consequences of redundancy and synergy during information transmission, suggesting a rationale for the regulation of these information processing features.

**DOI:** http://dx.doi.org/10.7554/eLife.24040.001

## Introduction

Signaling pathways convey information about the environment, enabling organisms to generate appropriate physiological response to changing conditions (Gendron et al., 2015). We recently established that *tph-1* tryptophan hydroxylase expressed in ADF and NSM neurons and *daf-7* TGFβ expressed in ASI neurons in *Caenorhabditis elegans* transmit environmental information to physiology by modulating the response of lifespan to food (Entchev et al., 2015). Our previous analytical framework estimated the accuracy of *tph-1* and *daf-7* expression in decoding food input; however, it could not reveal the type of encoding strategy used by *tph-1* and *daf-7* within these neurons, nor could it quantify the contribution of these genes to lifespan modulation. Here, we applied information theory (Shannon, 1948) to address these issues. Information theory has been proposed as a general framework to characterize how biological signals are encoded and transmitted (Bowsher and Swain, 2014; Levchenko and Nemenman, 2014) and has been used to study information processing in the nervous system (Borst and Theunissen, 1999) as well as biochemical and genetic pathways (Cheong et al., 2011; Tkačik et al., 2015).

Groups of neurons can encode information redundantly or synergistically (Brenner et al., 2000; Puchalla et al., 2005). This form of informational redundancy is conceptually distinct from genetic redundancy. Redundant encoding systems replicate the same information in more than one neuron, analogous to a computer backup, which provides robustness to perturbations in single neurons at the expense of coding efficiency. In contrast, synergistic circuits encode more information than the sum of their component neurons, but this efficiency is vulnerable to disruptions in the constituent neurons. Redundancy and synergy have been defined using information-theoretic measures (Averbeck et al., 2006; Schneidman et al., 2003), and both of these strategies for encoding information have been characterized in many neural and genetic circuits (Averbeck et al., 2006; Puchalla et al., 2005; Schneidman et al., 2011; Tkačik et al., 2015; Tkačik and Walczak, 2011).

Previously, we identified regulatory interactions among *tph-1* and *daf-7* that influence their coding accuracy (Entchev et al., 2015). Here, we show that cross-talk between *daf-7* and *tph-1* further affects the adoption of redundancy or synergy during discrimination between food levels. We found that the regulation of signal-to-noise in gene expression underlies shifts between redundancy and synergy across genotypes. Finally, we use a computational model to explore the consequences of redundant and synergistic coding at the level of downstream targets.

## Results and discussion

**Figure 1.**
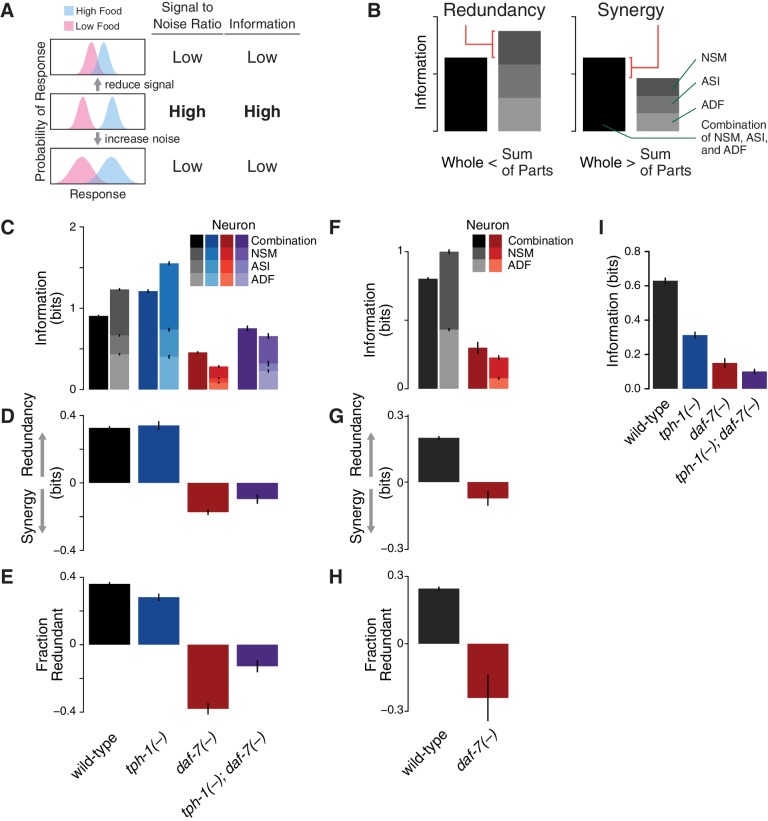
Redundancy and synergy in a gene expression code. (**A**) Information content depends on the overlap between gene expression distributions under different environmental conditions, which in turn depends on both the response magnitude (signal) and the variability across the population (noise). (**B**) Diagrams illustrating redundancy versus synergy, calculated as the difference between the whole (combinatorial information in NSM/ASI/ADF; darkest bar) and the sum of parts (information in NSM + ASI + ADF; stacked bars). (**C**–**E**) Analysis of redundancy and synergy based on *tph-1* expression in ADF and NSM, and *daf-7* expression in ASI. Genotype color key: Wild-type (black), *tph-1(-)* (blue), *daf-7(-)* (red), and *tph-1(-); daf-7(-)* (purple). (**C**) Effect of *tph-1(-)* and *daf-7(-)* mutations on food encoding in the whole circuit (darkest bars) and the sum of parts (lighter stacked bars). (**D**) Effect of *tph-1(-)* and *daf-7(-)* on redundancy and synergy among ADF, NSM, and ASI, as defined in Equation 2 and (**B**). As described in Equation 2 and in the main text, redundancy and synergy are indicated by positive and negative R values, respectively. (**E**) Fraction of redundant or synergistic information in ADF, NSM, and ASI, which is the amount of redundancy or synergy in (**D**) normalized to the information encoded. (**F**–**H**) Analysis of redundancy and synergy only in the *tph-1* expressing neurons, ADF, and NSM. (**F**) Effect of *daf-7(-)* in the information encoded by *tph-1* expression in ADF and NSM (darkest bars) and the sum of their parts (lighter stacked bars). (**G**) Effect of *daf-7(-)* on redundancy/synergy of ADF and NSM. (**H**) Fraction of redundant or synergistic information in *tph-1* expression in ADF and NSM, which is the amount of redundancy or synergy in (**G**) normalized to the total information encoded from (**F**). (**I**) Loss of *tph-1* and *daf-7* degrades information about food abundance at the level of lifespan responses. **DOI:** http://dx.doi.org/10.7554/eLife.24040.002 10.7554/eLife.24040.003Figure 1—source data 1.Information and redundancy across genotypes.(**Tab 1**) Combinatorial mutual information in the NSM/ASI/ADF neural circuit (‘Whole’ column) and the individual mutual information in ADF, ASI and NSM neurons across different genotypes. (**Tab 2**) Combinatorial information in the NSM and ADF neurons (‘Whole’ column) and the individual information in ADF and NSM neurons in wild-type and *daf-7(-)* strains. (**Tab 3**) Mutual information in the lifespan response of different genotypes. All values are presented as bits ± error.**DOI:** http://dx.doi.org/10.7554/eLife.24040.003 10.7554/eLife.24040.004Figure 1—source data 2.Fluorescence values for animals carrying both *Pdaf-7::mCherry* and *Pdaf-7::Venus* across four food levels for Figure 1—figure supplement 2.**DOI:** http://dx.doi.org/10.7554/eLife.24040.004 10.7554/eLife.24040.005Figure 1—source data 3.Optimal input distributions for ADF, ASI and NSM neurons across genotypes (data for Figure 1—figure supplement 3).Optimal input distributions obtained by maximizing the information encoded individually by ADF, ASI, and NSM neurons. Values are presented as probabilities ± uncertainty.**DOI:** http://dx.doi.org/10.7554/eLife.24040.005 10.7554/eLife.24040.006Figure 1—source data 4.Validation of information and redundancy estimates for Figure 1—figure supplement 4.(**Tab 1**) Information (MI/channel capacity) and redundancy encoded by food-responsive gene expression in wild-type animals computed using different methodologies for density estimation. From left to right: plug-in method, least squares cross-validation, and smoothing cross-validation (kernel density estimation with fixed bandwidth selection), balloon estimator (kNN), Jack-knife correction of sample size bias. (**Tab 2**) Jack-knife analysis for information and redundancy across all genotypes. Values are calculated using a fraction of the total dataset indicated in first column.**DOI:** http://dx.doi.org/10.7554/eLife.24040.006 10.7554/eLife.24040.007Figure 1—source data 5.Information, redundancy, and optimal input distribution by food level across genotypes.Data for Figure 1—figure supplement 5. Mutual information of ADF, ASI, and NSM neurons, redundancy and optimal input distribution of the whole circuit by food level across genotypes.**DOI:** http://dx.doi.org/10.7554/eLife.24040.007

Information theory allows us to quantify the information encoded by *daf-7* and *tph-1* based on the overlap of their expression distributions (Figure 1A). By associating environmental stimuli (food level) and neuronal responses (gene expression) with the input and the output of a communication system, the encoding capacity of ASI, ADF, and NSM is given by the mutual information (MI) between gene expression responses (G) and food stimuli (F),

(1) $$MI\left(G;F\right)=\sum_{G,F}P\left(F\right)P\left(G|F\right)\log\frac{P\left(G|F\right)}{P\,\left(G\right)}$$

where P⁢(F) denotes the chances of encountering the food condition F, P(G|F) is the response under each specific food level, and P⁢(G) is the average response across all the food stimuli (see Appendix and Figure 1—figure supplement 5). The MI measures the ability of the gene expression response to discriminate between food conditions.

**Figure 1—figure supplement 1.**
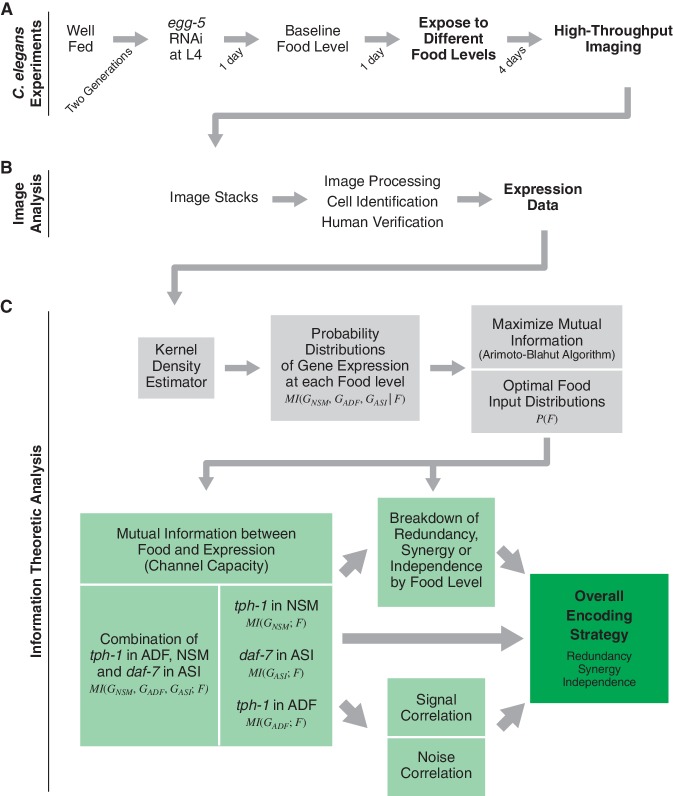
Schematic of experimental and analytical workflow. (**A**) Experimental procedure for imaging gene expression responses to different food levels in adult *C. elegans*. Animals carrying fluorescent reporters were cultured and exposed to six food levels. A custom microfluidics-based platform was used for quantitative high-throughput imaging of the reporters. (**B**) Image analysis pipeline to identify individual neurons and quantify their fluorescence. (**C**) Information theoretic analysis for dissecting coding strategy in multicellular gene expression circuits. We first used a kernel density estimator to obtain gene expression response probabilities from our data. Next, we obtained theoptimal food distributions and the maximal mutual information between food stimuli and gene expression response. This analysis highlights the relationships between several parameters that describe the multi-neuron gene expression responses (light green boxes) and their contributions to the overall encoding strategy (dark green box). **DOI:** http://dx.doi.org/10.7554/eLife.24040.008

**Figure 1—figure supplement 2.**
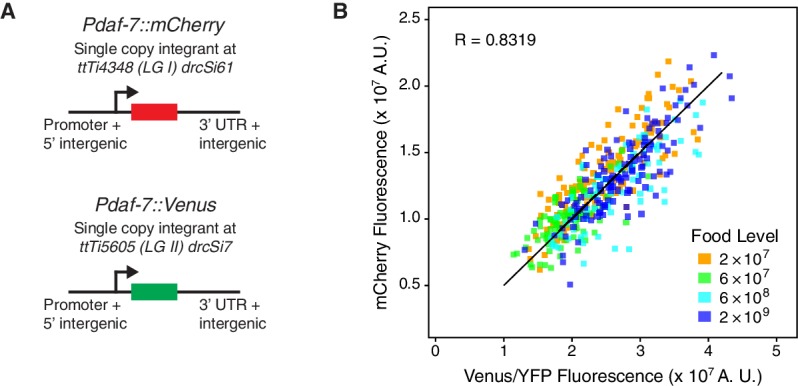
Experimental variability. (**A**) Animals carrying *Pdaf-7::mCherry* and *Pdaf-7::Venus* at different genomic locations were used to estimate experimental variability. (**B**) The strain described in (**A**) was shifted to four different food levels (legend) and then imaged simultaneously for mCherry and Venus fluorescence. The graph shows a good correlation between mCherry and Venus reporter expression (R=0.8319). A total of 400 animals were imaged in this experiment. **DOI:** http://dx.doi.org/10.7554/eLife.24040.009

**Figure 1—figure supplement 3.**
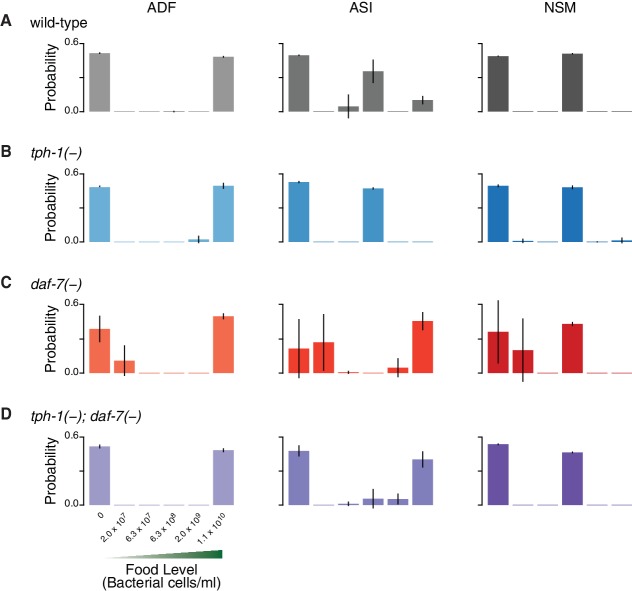
Neurons differ in their optimal input distributions. Optimal input distributions obtained by maximizing the information encoded individually by ADF, ASI and NSM neurons qualitatively differ. This feature may allow different neurons to detect different food input levels to broaden the sensory range of the whole circuit. The optimal input distribution for each neuron also differ by genotype: (**A**) wild-type, (**B**) *tph-1(-)* mutants, (**C**) *daf-7(-)* mutants, and (**D**) *tph-1(-); daf-7(-)* double mutants. Uncertainties are obtained from sampling the 80% of the data and taking the standard deviation. **DOI:** http://dx.doi.org/10.7554/eLife.24040.010

**Figure 1—figure supplement 4.**
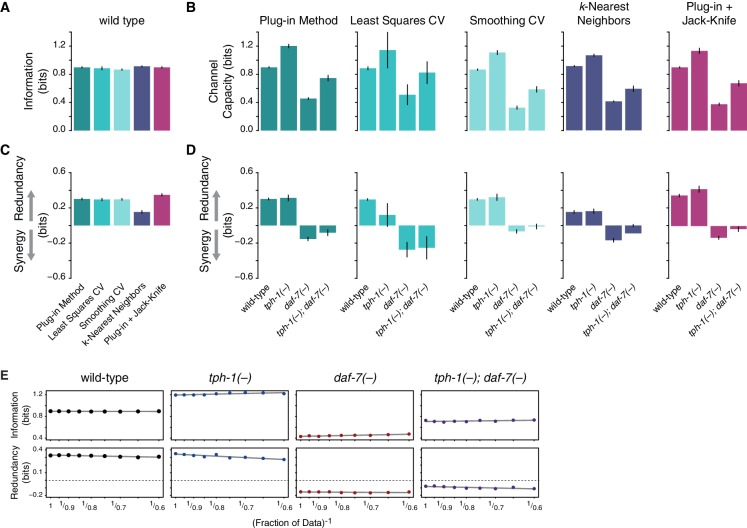
Robustness of information theoretic analyses. (**A**) Information (MI/channel capacity) encoded by food-responsive gene expression in wild-type animals computed using different methodologies for density estimation. From left to right: plug-in method, least squares cross-validation, and smoothing cross-validation (kernel density estimation with fixed bandwidth selection), balloon estimator (kNN), Jack-knife correction of sample size bias. All this methods result in similar information values. (**B**) Information encoded by food-responsive gene expression in wild-type, *tph-1(-)*, *daf-7(-)*, and *tph-1(-); daf-7(-)* animals. The relative changes in information between different approaches do not display significant differences. (**C**) and (**D**) illustrate the same analysis for redundancy. The switch from redundancy in wild-type to synergy in *daf-7(-)* is consistently present for all the methodologies used. (**E**) Details of jack-knife analysis for information and redundancy across all genotypes. Information and redundancy values are calculated using a fraction of the total data indicated in the x-axis. Dashed line (bottom) indicates a redundancy value of zero, separating redundancy and synergy. Both information and redundancy are stable to the sample size, as indicated by the flat lines of best fit. Error bars are standard deviation derived from sampling 80% of the data. **DOI:** http://dx.doi.org/10.7554/eLife.24040.011

**Figure 1—figure supplement 5.**
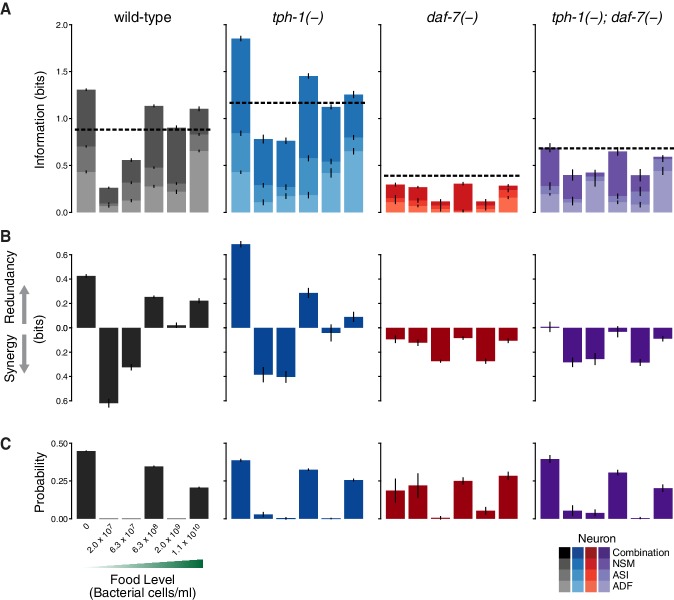
Information and redundancy by food level. (**A**) The sum of information encoded by ADF, ASI, and NSM for each food level across different genotypes is indicated by stacked bars. Information in each neuron is indicated by the legend (bottom right). Dashed lines indicate the information encoded by the combinatorial gene expression in the whole circuit, which is constant across food levels (see Supplemental Materials and methods for mathematical details). (**B**) Redundancy values across food levels for each genotype. (**C**) The optimal distribution of food input that maximizes information encoded by the whole circuit. **DOI:** http://dx.doi.org/10.7554/eLife.24040.012

To define the redundancy of the system (Schneidman et al., 2003), we considered the difference between the sum of the information independently encoded by gene expression in the ADF, ASI, and NSM neurons, and the MI obtained from their combinatorial expression (Figure 1B):

(2) $$R=\underset{s\,u\,m\,o\,f\,p\,a\,r\,t\,s}{\underbrace{M\,I\,\left(G_{A\,D\,F};F\right)+M\,I\,\left(G_{A\,S\,I};F\right)+M\,I\,\left(G_{N\,S\,M};F\right)}}-\underset{w\,h\,o\,l\,e}{\underbrace{M\,I\,\left(G_{A\,D\,F},G_{A\,S\,I},G_{N\,S\,M};F\right)}}$$

Conceptually, redundancy occurs when the whole is less than the sum of parts (R>0), whereas synergy occurs when the whole is greater than the sum of parts (R<0) (Figure 1B).

This analysis revealed that ASI, ADF, and NSM neurons encode ∼0.9 bits of information about food abundance in wild-type animals (Figure 1C), which is in the same range of information encoded by other biochemical pathways (Cheong et al., 2011), and it is consistent with the requirement for sensing the two states (boom or bust) experienced by *C. elegans* in the wild (Félix and Braendle, 2010). Approximately 40% of this information is encoded redundantly in wild-type animals (Figure 1D–E), consistent with the genetic evidence that *tph-1* and *daf-7* act in parallel pathways to modulate lifespan (Entchev et al., 2015). *tph-1(-)* and *daf-7(-)* mutants show respective increases and decreases in food information (Figure 1C), consistent with our prior decoding analysis. *tph-1(-)* mutants also show a modest decrease in the fraction of redundant information (Figure 1E), suggesting that the added information is more efficiently but less robustly encoded.

Remarkably, changes in the expression distributions of the *daf-7* and *tph-1* reporters in *daf-7(-)* mutants shift the encoding strategy of ASI, ADF, and NSM from redundancy to synergy (Figure 1C–D), such that ∼40% of the total information in the circuit is now encoded synergistically (Figure 1E). This effect is not due to the loss of ASI function in *daf-7(-)* mutants, as we observed the same shift to synergy when only *tph-1(-)* expressing neurons are analyzed (Figure 1F–H), indicating that crosstalk between *daf-7* and *tph-1* as well as *daf-7* autoregulation control the coding strategy adopted by the circuit. Importantly, the coding strategy shift is *daf-7-*specific, as disruption of *tph-1* does not result in a similar phenotype (Figure 1C). In the *tph-1(-); daf-7(-)* double mutant, cross- and self-regulation are abolished, and ASI, ADF, and NSM neurons approach the independence regime (R=0) (Figure 1C–E), confirming the idea that redundancy and synergy arise from the communication between neurons via *daf-7* and *tph-1*.

The same information-theoretic analysis can be applied to quantify more directly the contribution of *daf-7* and *tph-1* to the food-responsiveness of the physiological output. The lifespan response to food abundance consists of ∼0.6 bits of information in wild-type animals, and approximately 80% of this food information is lost in the *tph-1(-); daf-7(-)* double mutant (Figure 1I), strengthening our previous assertion that the majority of the food information encoded in the lifespan response is mediated by *tph-1* and *daf-7*. While other genetic pathways may also play important roles, this central role of *tph-1* and *daf-7* suggests that their coding features weigh heavily on the physiological outcome.

**Figure 2.**
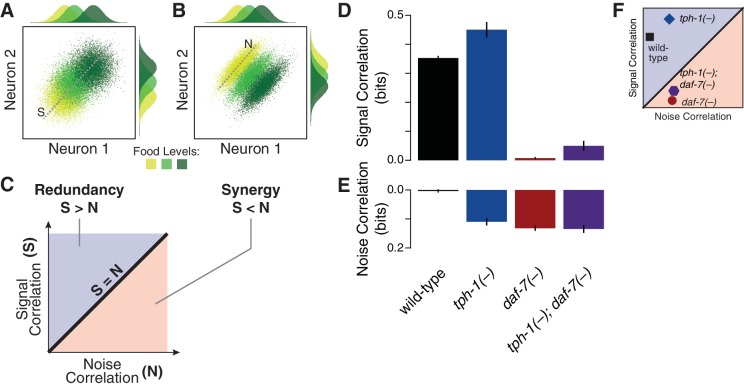
Signal and noise correlations influence redundancy and synergy. (**A**–**B**) Hypothetical expression distributions of two neurons at three food levels, illustrating signal and noise correlations and their effects on redundancy (Schneidman et al., 2003). Centre: their 2D distributions. Top and side: the distributions of each neuron. Signal correlation between two neurons across three food levels, and noise correlation at one selected food level are denoted by dotted lines marked ‘S’ and ‘N’ in (**A**) and (**B**), respectively. (**C**) shows how signal and noise correlations are related to redundancy and synergy as previously established (Schneidman et al., 2003). When signal correlations are higher (**A**), each neuron provides similar information (top and side distributions), reflecting redundancy. When noise correlations are higher (**B**), the combinatorial expression shows reduced overlaps and contains more information than individual neurons, providing synergy. (**D**–**E**) The effects of *daf-7* and *tph-1* on redundancy and synergy are explained by their effects on the signal correlation (**D**) and noise correlation (**E**). (**F**) Signal and noise correlation in each genotype and their relation to redundancy and synergy as indicated in (**C**). **DOI:** http://dx.doi.org/10.7554/eLife.24040.013 10.7554/eLife.24040.014Figure 2—source data 1.Signal and noise correlations across genotypes.**DOI:** http://dx.doi.org/10.7554/eLife.24040.014

Multicellular coding strategies rely on response correlations between cells (Schneidman et al., 2003). Specifically, redundancy can be dissected into two components: the signal correlation, which reflects correlated average responses (Figure 2A) and increases redundancy; and the noise correlation, which captures co-fluctuations among different cells under fixed food levels (Figure 2B–C) and promotes synergy (Schneidman et al., 2003) (Appendix). As opposed to the wild-type animals, where the negligible value of noise correlation leads to redundancy (Figure 2D–E), all mutants display a general increase of noise correlations. *tph-1(-)* animals retain redundancy by compensating this effect with an increase of signal correlation; however, this balance shifts in the *daf-7(-)* mutant due to the dramatic reduction of signal correlation (Figure 2F), bringing the system to the synergistic regime (Figure 1D). The *tph-1(-); daf-7(-)* double mutant has nearly equal signal and noise correlations which generate independent encoding.

**Figure 3.**
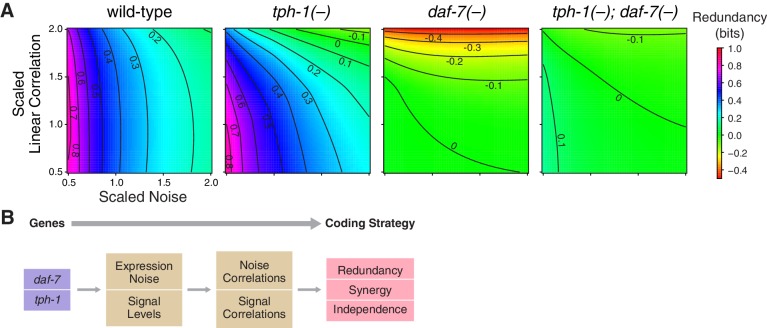
Interplay between noise and correlation affects redundancy. (**A**) Heat maps showing redundancy when correlation and noise are scaled from their baseline values in wild-type and mutants. Redundancy values are indicated by legend. Contour lines denote equal redundancy. The number of contour lines crossed along each axis indicates the sensitivity to that parameter. (**B**) The steps leading from genes to coding strategy. **DOI:** http://dx.doi.org/10.7554/eLife.24040.015

Redundancy and synergy is strongly affected by noise and correlation among neurons. To characterize their effects, we rescaled noise and correlations in the original response distributions of *daf-7* and *tph-1* over a biologically relevant range (Figure 3, Appendix). In wild-type animals, redundancy is highly sensitive to noise, and weakly sensitive to correlation, providing a rationale for *daf-7* in noise reduction (Entchev et al., 2015). *tph-1(-)* mutants displayed increased sensitivity to both noise and correlations. Redundancy in *daf-7(-)* mutants was more sensitive to correlation than noise, a reversal of the wild-type situation. *tph-1(-); daf-7(-)* double mutants were less sensitive to noise and correlations than either single mutant. These results suggest that the sensitivity of redundancy to noise is controlled by *daf-7*, while robustness to correlation is maintained by both *daf-7* and *tph-1*.

**Figure 3—figure supplement 1.**
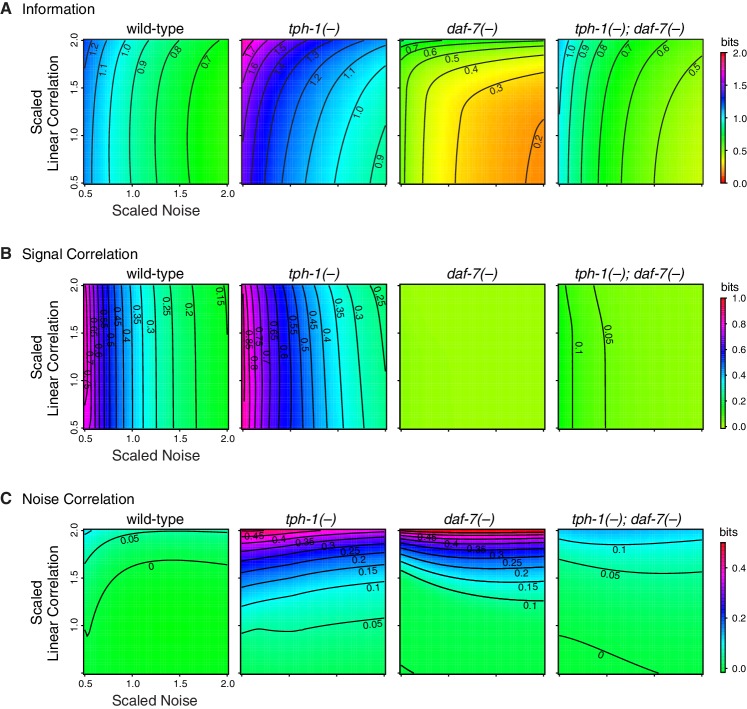
Sensitivity analysis of channel capacity, signal, and noise correlation. Heat maps showing how channel capacity (**A**), signal correlation (**B**) and noise correlation (**C**) vary under a rescaling of the baseline covariance matrix. For this analysis, response distributions were modeled using multivariate normal distributions (see Appendix). **DOI:** http://dx.doi.org/10.7554/eLife.24040.016

**Figure 4.**
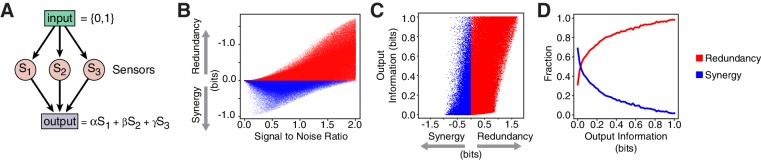
Computational model reveals advantages of redundancy. (**A**) Model for information encoding and transmission, where three sensors activate one target that integrates their signals linearly (see Appendix). (**B**) Effect of signal-to-noise ratio on coding strategy. (**C**) Effect of coding strategy on transmitted information. (**D**) Sensors that transmit more information tend to use redundancy. **DOI:** http://dx.doi.org/10.7554/eLife.24040.017

Redundancy or synergy in *daf-7* and *tph-1* expressing neurons serves as one constraint but does not necessarily lead to the same coding strategy in their targets. The coding strategy used by these targets will depend on their connectivity to ASI, ADF, and NSM, as well as their noise, correlation, and dynamic range. Since little is known about the immediate targets of TGFβ and serotonin signaling in relation to the food response in *C. elegans*, we considered a minimal model of three ideal sensors detecting an input and transmitting to a target that integrates linearly their signals (Figure 4A, Appendix). This simple model shows that decreasing signal-to-noise ratio favors synergy (Figure 4B, Appendix), in agreement with the observation that *daf-7(-)* mutants show reduced signal-to-noise, and adopt synergistic encoding (Figure 1D–F). This model also explains the decrease in synergy in *tph-1(-); daf-7(-)* double mutants compared to *daf-7(-)* single mutants (Figure 1D–F): loss of *tph-1* increases signal separation (Entchev et al., 2015), which increases signal-to-noise, thus reducing synergy. Thus, that signal-to-noise ratios can contribute significantly to the coding strategy.

**Figure 4—figure supplement 1.**
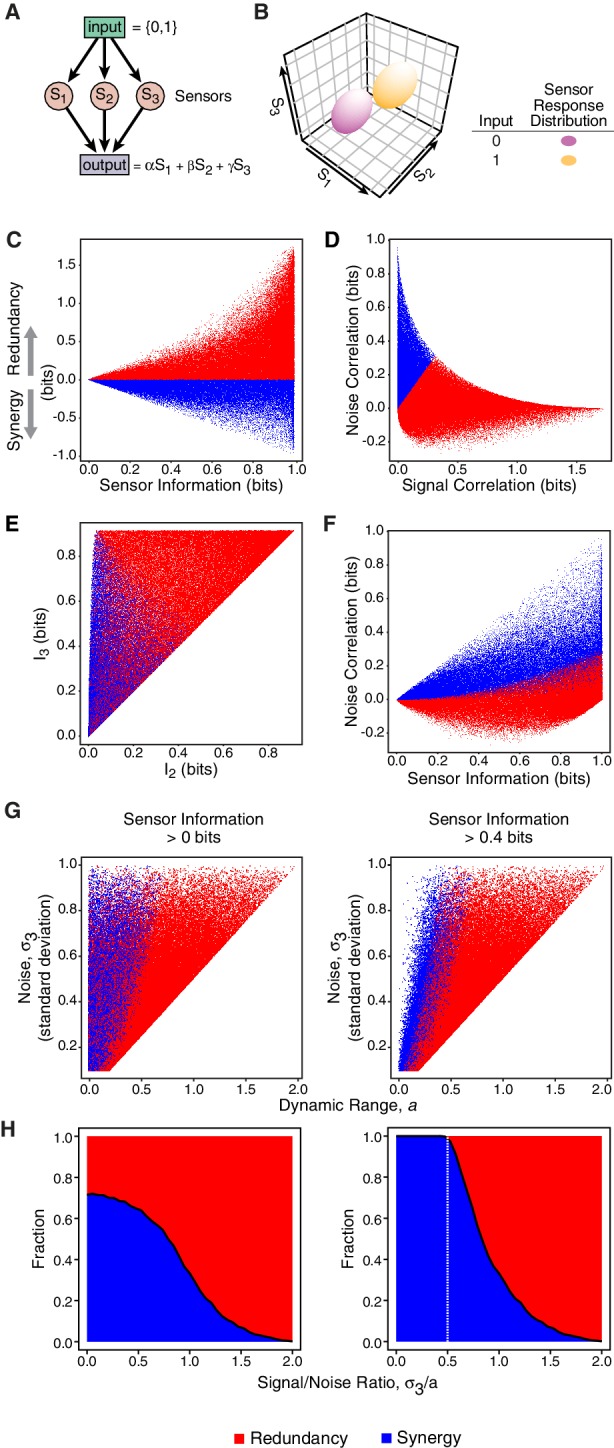
Gaussian model of sensory neurons and information transmission. (**A**) Illustration of the model. $S_{k}$ represent single sensors receiving information from the binary input. The sensor response is then combined linearly by the output. (**B**) The joint sensor response distribution is modeled as a three-dimensional Gaussian centered at input-dependent average values. (**C**, **D**, **E**, **F**) display the result of the numerical analysis of the model. Each point represents a specific choice of the parameters used in the model. For all sampled parameter sets (N=500000), we obtained the relevant information-theoretic measures shown. Red and blue colors are used to distinguish redundant and synergistic regimes, respectively. (**G**) The sampled conditions sliced according to dynamic range (a) and noise ($\sigma_{3}$) (left). Redundant configurations populating the low signal-to-noise ratio ($S\,N\,R=a/\sigma_{3}$) provide typically low information. S⁢N⁢R becomes a discriminant between redundancy and synergy when applying a non-zero cutoff to the information encoded by the sensors (right). (**H**) Fraction of redundant/synergistic configurations obtained by in the numerical exploration of the model (left). When sensors are required to encode a minimum level of information, parametric configurations with low S⁢N⁢R are forced to be synergistic, providing a rational for the coding strategy switch observed in *daf-7(-)*. **DOI:** http://dx.doi.org/10.7554/eLife.24040.018

Our model also illustrates the advantages of redundancy in the case of linear integration. Redundant strategies increase the minimum information transmitted to a downstream target when compared to a synergistic encoding (Figure 4C). Additionally, redundant encoding not only allows higher information transmission, but can also be accommodated by a broader set of signaling parameters (Figure 4D), avoiding the need to fine tune biological properties. When considering lifespan as the downstream target, our model suggests that lifespan responsiveness to food should decrease in *daf-7(-)* mutants, because wild-type animals employ redundancy, whereas *daf-7(-)* mutants employ a synergistic encoding. Indeed, we find that the ability to accurately discriminate between different food inputs based on lifespan is degraded in *daf-7(-)* mutants (Figure 1I) (Entchev et al., 2015).

By extending the analysis of our previous work, we have found that the ADF, NSM and ASI neurons employ a redundant strategy to encode food information. Critically, this redundant encoding strategy is controlled by *daf-7* TGFβ and modified by *tph-1* tryptophan hydroxylase; this is a novel effect of neuromodulators on circuit function. In particular, we revealed two roles for *daf-7*: as an encoder of food information, and as a regulator of redundancy via regulation of *tph-1*. In principle, redundancy and synergy could be specified by many different biological mechanisms, with obvious candidates being developmental changes in sensor types or numbers in a neural circuit. These mechanisms are ruled out in *daf-7(-)* and *tph-1(-)* animals, as the mutations do not affect the development of the ASI, ADF, and NSM neurons, which remain food-responsive. Instead, we show that *daf-7* and *tph-1* influence information processing via effects on the signal and noise properties of these sensory neurons, and on their correlations, representing additional roles for these genes in controlling information encoding. The discovery of other genes that regulate the signal-to-noise ratio will likely provide further insights into genetic regulatory mechanisms that modulate neural coding.

## Computational methods

### Minimization and quantification of experimental noise

Information theory relies on accurate estimates of response distributions, requiring the minimisation of experimental variability. We took several steps to achieve this. First, we only considered animals oriented in a dorso-ventral position. The microfluidic chip was constructed to bias animals towards this correct orientation, the orientation was checked during automated cell identification and verified manually, ensuring that only image stacks with animals in dorso-ventral orientations were used in the analysis. Second, we used direct imaging of transcriptional fusions to fluorescent protein reporters integrated in single copy. This approach ensures that biological variance in promoter activity is not artificially washed out by averaging in conventional high-copy reporters that are more traditionally used to generate *C. elegans* transgenics. Using fluorescent reporters also eliminates experimental noise associated with antibody staining due to variability in fixation, in permeabilizing the *C. elegans* cuticle, and in signal amplification from secondary antibodies. Third, we minimized bleaching by using a combination of low excitation from an LED light source, and rapid image acquisition using a Piezo Z stage (Prior Scientific) that precisely moves the sample in the Z axis at high speed.

In addition, we used simultaneous quantification of mCherry and Venus/YFP driven by the same promoter to estimate our experimental noise (Figure 1—figure supplement 2). We generated animals with *Pdaf-7::mCherry* and *Pdaf-7::Venus* reporters integrated at single copy in precise genomic locations on LG I and LG II, respectively (Figure 1—figure supplement 2A). These animals were shifted to four different food levels and imaged 1 day after the food shift. This experimental measurement incorporates experimental noise associated with different fluorescent proteins (mCherry and Venus) and different chromosomal locations for reporters, as well as other methodological noise. We found that the two measurements were in good agreement (R∼0.83, Figure 1—figure supplement 2B). Dissecting the variance in these measurements showed that 30% ($1-R^{2}$) of the observed variability in these measurements was due to variability between the mCherry and Venus readouts. We note that this variability includes intrinsic noise as the reporters are on different chromosomes; the actual experimental variability would therefore be lower, since intrinsic noise is non-zero.

### Computational analysis

The computational analysis of all the data was performed using custom-made C++ programs and built-in implementations of standard multivariate analysis algorithms in R (R Core Team, 2016). C++ programs are available through GitHub repositories (https://github.com/giovannidiana/Information, https://github.com/giovannidiana/KDE and https://github.com/giovannidiana/ModelRS). Mathematical details of these procedures and the results are discussed in the Appendix.

## Appendix

Supplementary computational methods To uncover the information processing features of the *daf-7*/*tph-1* genetic circuit embedded in the ASI, ADF and NSM neurons, we performed an information-theoretic analysis of the gene expression responses of *daf-7* in the ASI neuron, and *tph-1* in the ADF and NSM neurons. In the main text we introduced the mutual information (MI) as a measure of the correlation between food level and gene expression. In this section we discuss in greater detail the properties of this quantity and the procedure used to estimate MI from our gene expression data. The type of encoding system that we are interested in maps an input F (food level) taking $N_{F}=6$ distinct values $\left\{f_{1},\cdots ,f_{N_{F}}\right\}$ onto three continuous variables denoted by the vector $G=\left\{G_{A\,D\,F},G_{A\,S\,I},G_{N\,S\,M}\right\}$ (gene expression in the three neurons). Mutual information The multivariate gene expression response under a specific food condition is given by the set of conditional probabilities $P\left(G|F=f_{k}\right)$, ($k=1,\ldots ,N_{F}$). To characterize the information transmission of a communication system we also need to specify the probabilities P⁢(F) with which the ADF-ASI-NSM encoder is exposed to each food condition. Given the input probabilities P⁢(F) we can compute averages across food level, in particular the marginal probabilities of gene expression

(3) $$P\left(G\right)=\sum_{k=1}^{N_{F}}P\left(f_{k}\right)P\left(G|f_{k}\right).$$

From input and response distributions we can build up three information entropies. First, the joint information entropy of both food and gene expression is defined as (Cover and Thomas, 2006)

(4) $$H\,\left(F,G\right)=\int d^{3}\,G\,\sum_{k=1}^{N_{F}}P\,\left(G,F\right)\,\log_{2}P\,\left(G,F\right),$$

and it measures the variability of input and output. Second, we can quantify the variability of the gene expression response to food by the conditional entropy

(5) $$H\left(G|F\right)=\int d^{3}G\sum_{k=1}^{N_{F}}P\left(G,F\right)\log_{2}P\left(G|F\right),$$

Third, the entropy of the marginal distributions in Equation (3)

(6) $$H\,\left(G\right)=\int d^{3}\,G\,P\,\left(G\right)\,\log_{2}P\,\left(G\right),$$

measures the variability of the average response. The mutual information defined as

(7) $$\begin{array}{ll} MI(G;F) & =\int d^{3}G\sum_{k=1}^{N_{F}}P(G,f_{k})\log_{2}\frac{P(G,f_{k})}{P(G)P(f_{k})}=\\ & =\int d^{3}G\sum_{k=1}^{N_{F}}P(f_{k})P(G|f_{k})\log_{2}\frac{P(G|f_{k})}{P(G)}\geq 0. \end{array}$$

is always positive due to the log-sum inequality and it can be expressed as the difference between the gene expression entropy and the conditional entropy with respect to food level, that is

(8) MI(G;F)=H(G)-H(G|F)

which yields to the standard interpretation of M⁢I as the amount of information entropy shared between stochastic variables. As mentioned in the main text and Figure 1A, the mutual information is strongly affected by the signal-to-noise ratio (S⁢N⁢R). For univariate distributions, we use the definition

(9) $$S\,N\,R\equiv \sqrt{\frac{\mathrm{var}\left(G\right)-{\left\langle \mathrm{var}\left(G|F\right)\right\rangle }_{F}}{{\left\langle \mathrm{var}\left(G|F\right)\right\rangle }_{F}}},$$

where var(G|F) is the variance under the condition F and ${\left\langle \cdot \right\rangle }_{F}$ denotes the average across all conditions. MI can be decomposed as

(10) $$MI(G;F)=\sum_{k=1}^{N_{F}}P(f_{k})D_{k}^{\left(joint\right)}$$

where the components $D_{k}^{\left(j\,o\,i\,n\,t\right)}$ are defined as

(11) $$D_{k}^{\left(j\,o\,i\,n\,t\right)}\equiv D\left(P\left(G|f_{k}\right)||P\left(G\right)\right)=\int d^{3}GP\left(G|f_{k}\right)\log_{2}\frac{P\left(G|f_{k}\right)}{P\,\left(G\right)}$$

and represent the relative entropy between conditional and average response. Our estimates of mutual information provide a lower bound of the true information encoded by *tph-1* and *daf-7* due to noise inherent in all experiments. Channel capacity A common question in biology is to understand how phenotypic changes are related to the environmental input. To address this question it is natural to design experiments where relevant input variables are controlled. These types of experiments provide a good sampling of the responses, but the frequencies of environmental conditions at which biological systems are exposed in the wild are not always known. On the other hand, the level of information encoded about the environment depends on the input distribution. A common procedure to infer the input distribution is to assume that the set of gene expression responses is designed to maximize the information stored (Tkačik and Walczak, 2011; Selimkhanov et al., 2014; Uda et al., 2013). With this assumption we can obtain the food distribution by maximising the mutual information between food and gene expression. The maximal MI achievable given the set of conditional responses is known as channel capacity

(12) $$C=\max_{P\,\left(F\right)}\left(M\,I\,\left(G;F\right)\right),$$

and it is an intrinsic property of the encoding system. An important aspect of MI is that all the relative entropies $D_{k}^{\left(j\,o\,i\,n\,t\right)}$ in the decomposition (10) become identical under the optimality condition, which is easy to prove by maximizing the action

(13) $$S=H\left(G\right)-H\left(G|F\right)+\lambda \sum_{k}P\left(f_{k}\right)$$

where λ is a Lagrange multiplier to assure the normalization of the input distribution. By considering the derivative over $P\,\left(f_{k}\right)$ we get

(14) $$0=\frac{\delta \,S}{\delta \,P\,\left(f_{k}\right)}=D_{k}^{\left(j\,o\,i\,n\,t\right)}-1+\lambda$$

which implies that at the maximum, all the $D_{k}^{\left(j\,o\,i\,n\,t\right)}$ are equal to 1-λ. Therefore, since the channel capacity is defined as an average of $D_{k}^{\left(j\,o\,i\,n\,t\right)}$, $D_{k}^{\left(j\,o\,i\,n\,t\right)}=C$ for all k. This property implies that the optimal input distribution obtained by maximizing MI is such that all the conditional responses are equally distant from their average, when relative entropy is used as a measure of distance between probability distributions. In Figure 1—figure supplement 5A we compare the channel capacity obtained from *tph-1*/*daf-7* expression in ADF, ASI and NSM neurons (dotted line) with the components $D_{k}^{\left(n\,e\,u\,r\,o\,n\right)}$ of the mutual information between gene expression and food abundance in each neuron (ADF, ASI, NSM) for all genetic backgrounds. For this comparison we first obtained the channel capacity and the optimal input distribution from the three-dimensional data for each genotype (Figure 1—figure supplement 5C) and then we used this optimal distribution to calculate the mutual information of each neuron. The components of the mutual information for individual neurons are obtained from Equation (11) by using the corresponding marginal distribution. Unlike the components $D_{k}^{\left(j\,o\,i\,n\,t\right)}$ of the maximized joint mutual information, the $D_{k}^{\left(n\,e\,u\,r\,o\,n\right)}$ are not constant over the food level, reflecting the fact that single neurons are optimized for different input distributions (Figure 1—figure supplement 3). The optimal input frequencies (Figure 1—figure supplement 5C) reveal that wild-type animals encode the most information when they are most likely to encounter the highest, an intermediate, and the lowest food levels. This result implies that wild-type animals are best at detecting these food levels, compatible with the boom and bust lifestyle of *C. elegans* in the wild (Félix and Braendle, 2010). This optimal is altered in *daf-7(-)* mutants (Figure 1—figure supplement 5C), indicating that it is genetically controlled. By maximizing the mutual information between individual neurons and food conditions we find that each neuron is specialized to sense different food levels (Figure 1—figure supplement 3), which broadens their combined range of detectable food levels. For example, *tph-1* expression in ADF in wild-type animals is best at detecting the food extremes (Figure 1—figure supplement 3A). At these extreme food levels, ADF carries more information than at other food levels. Thus specialization among food sensing neurons ultimately leads to food-dependent heterogeneity in coding (Figure 1—figure supplement 5A). We note that the switch from redundancy in wild-type to synergy in *daf-7(-)* mutants still occurs when we use the wild-type optimal input frequency for calculating redundancy values for *daf-7(-)* mutants. Thus, our conclusions are not sensitive to the choice of using channel capacity and the corresponding optimal input distribution for each genotype. All the estimates of channel capacity in this work were done by using the standard Arimoto-Blahut algorithm (Arimoto, 1972; Blahut, 1972). Redundancy and synergy In the main text we introduce redundancy R as the difference (Schneidman et al., 2003)

(15) $$R=M\,I\,\left(G_{A\,D\,F};F\right)+M\,I\,\left(G_{A\,S\,I};F\right)+M\,I\,\left(G_{N\,S\,M};F\right)-M\,I\,\left(G;F\right)$$

and synergy as the negative of the redundancy. By following the work of Schneidman *et al.* the redundancy can be written as the difference between signal and noise correlation defined as

(s) (16)SC=MI(GADF;F)+MI(GASI;F)+MI(GNSM;F)−I(s)(17)NC=MI(G;F)−I(s)

where $I^{\left(s\right)}$ is the ‘shuffle’ information defined as

(18) $$I^{\left(s\right)}=\int d^{3}G\sum_{k}P\left(f_{k}\right)\cdot P^{\left(s\right)}\left(G|f_{k}\right)\log\frac{P^{\left(s\right)}\left(G|f_{k}\right)}{\sum_{k}P\left(f_{k}\right)P^{\left(s\right)}\left(G|f_{k}\right)},$$

and corresponds to a modified version of the mutual information between gene expression and food level where the joint distribution is replaced by the product of the marginal densities $P^{\left(s\right)}$,

(19) $$P^{\left(s\right)}\left(G|F\right)\equiv P\left(G_{A\,D\,F}|F\right)\cdot P\left(G_{A\,S\,I}|F\right)\cdot P\left(G_{N\,S\,M}|F\right).$$

By using the definition of the mutual information per neuron we can rewrite the signal correlation in the form of a relative entropy

(20) $$SC=\int d^{3}G\sum_{k}P\left(f_{k}\right)\cdot P^{\left(s\right)}\left(G|f_{k}\right)\log\frac{\sum_{k}P\left(f_{k}\right)P^{\left(s\right)}\left(G|f_{k}\right)}{P\,\left(G_{A\,D\,F}\right)\,P\,\left(G_{A\,S\,I}\right)\,P\,\left(G_{N\,S\,M}\right)}\geq 0$$

which shows that S⁢C is a non-negative quantity. As opposite to the signal correlation, which can only increase the level of redundancy, noise correlation can be positive or negative. Depending on the sign of R in Equation (15) the system operates in a redundant (S⁢C>N⁢C) or synergistic (S⁢C<N⁢C) regime. These information-theoretic measures of correlation reveal changes in different genotypes that contribute to shifts in coding strategy and capture different features of the interaction between the neurons. Consider for instance the case of independent encoders where the probability distribution of the joint response is factorized into the product of the responses of each neuron

(21) $$P\left(G|F\right)=P^{\left(s\right)}\left(G|F\right)=P\left(G_{A\,D\,F}|F\right)\cdot P\left(G_{A\,S\,I}|F\right)\cdot P\left(G_{N\,S\,M}|F\right).$$

In this case the noise correlation vanishes identically, however, signal correlation can be non-zero due to the correlation induced by the stimulus, thus we obtain the intuitive result that the level of redundancy in a system of independent encoders is always non-negative. Therefore, the synergistic encoding that we observe in the *daf-7(-)* mutant is caused by the change in the interaction network of ADF, ASI and NSM neurons. In the wild-type this network is tuned to guarantee a robust encoding of food abundance. When *daf-7* is knocked-out, the sign of R in Equation (15) changes, which namely corresponds to a switch from redundancy to synergy. Analogously to the mutual information, also redundancy can be decomposed as

(22) $$R=\sum_{k=1}^{N_{F}}P\,\left(f_{k}\right)\,D_{k}^{\left(r\,e\,d\right)}$$

where we defined the food components $D_{k}^{\left(r\,e\,d\right)}$ as

(23) $$D_{k}^{r\,e\,d}=D_{k}^{\left(A\,D\,F\right)}+D_{k}^{\left(A\,S\,I\right)}+D_{k}^{\left(N\,S\,M\right)}-D_{k}^{\left(j\,o\,i\,n\,t\right)}.$$

In Figure 1—figure supplement 5B we show the redundancy components at each food level across all genotypes. We observe that the quality of the encoding (synergistic or redundant) varies under different food conditions. In particular, both wild-type and *tph-1(-)* mutant tend to adopt a synergistic behaviour under non optimal food conditions (see Figure 1—figure supplement 3A–B for comparison with input distributions) whereas *daf-7(-)* mutant and *tph-1(-); daf-7(-)* double mutant are always synergistic. The input distribution obtained by maximizing $M\,I\,\left(G_{A\,D\,F},G_{A\,S\,I},G_{N\,S\,M};F\right)$ was used as a reference food distribution for all the genotypes analyzed in this work. *tph-1* and *daf-7* promoter activity was available also for mutant strains because the reporters were separate from the endogenous genes. To confirm that the synergistic character of the encoding in the *daf-7(-)* mutant is not an artifact of including ASI (where *daf-7* is expressed) in the estimation of the redundancy, we performed the same analysis by using only ADF and NSM read-outs. As a result, by comparing wild-type and *daf-7(-)* mutant, we obtained the same qualitative switch from redundant to synergistic encoding as obtained from the inclusion of all neurons (Figure 1F–H). Kernel density estimation Information entropies, and thus mutual information, are functionals of the probability distribution of the readouts. To quantify the conditional distributions P(G|F) we used Kernel Density Estimation (KDE) (Scott, 1992), which provides a mathematical framework to estimate distributions of continuous variables. Compared to the standard methodology of frequency histograms to estimate distributions, this technique does not require bin size selection. In the KDE approach, the probability density is estimated by the sum of reference distributions (kernel) centered at the observed values, thus for any expression vector g we have the estimated density $\hat{f}(g)$ reads

(24) $$\hat{f}(g)=\frac{1}{n}\sum_{i=1}^{n}K_{H}(g-G_{i})$$

where the kernel $K_{H}$ is a multivariate Gaussian distribution, the ‘bandwidth’ H corresponds to its variance matrix and the sum is over all the measured expressions ${\left\{G_{i}\right\}}_{i=1}^{n}$. An accurate estimation of the density relies on the choice of the bandwidth, which can be constant across the support of the probability or adapted to the local density. The Mean Squared Error (MSE)

(25) $$MSE[\hat{f}(g)]=E\{(\hat{f}(g)-P(g|F){)}^{2}\}=Var[\hat{f}(g)]+{Bias}^{2}[\hat{f}(g)]$$

and its integral (MISE) are commonly minimized to find the appropriate bandwidth. Selector algorithms differ in the trade-off between bias and variance of the estimator. To check the robustness of our calculation, we compared the results obtained by using different fixed bandwidth selector algorithms (Figure 1—figure supplement 4). In particular, we used the plug-in method (Chacón and Duong, 2010), least squares cross-validation (Bowman, 1984) and smoothing cross validation (Jones et al., 1991), all of which provide a uniform bandwidth. The general, fixed bandwidth estimators tends to oversmooth the main part of the distribution and undersmooth the tails. To confirm that this effect did not introduce artificial biases we also used the ‘baloon’ (k-nearest neighbours) estimator (Loftsgaarden and Quesenberry, 1965), where the probability distribution is proportional to the local density of observations (Figure 1—figure supplement 4). Once we obtained the conditional response distributions, averages over expression levels as in Equation (7) were computed by evaluating P(G|F) on a three-dimensional grid (a different approach would be to resample from the obtained distribution (Krishnaswamy et al., 2014). By testing different grid resolutions we found that a grid of size $30^{3}$ was sufficient to guarantee the convergence of averages. The uncertainty in the estimation of channel capacity was obtained by calculating the variance associated with sampling the 80% of the data. As shown in Figure 1—figure supplement 4, the estimates of both channel capacity and redundancy/synergy are robust to KDE algorithm in all genetic backgrounds. Sample size bias A well known issue in the estimation of channel capacity is the bias due to sample size. The general jack-knife procedure to remove this effect involves expanding the channel capacity in inverse powers of sample size (Cheong et al., 2011; Selimkhanov et al., 2014),

(26) $$C_{b\,i\,a\,s\,e\,d}=C_{u\,n\,b\,i\,a\,s\,e\,d}+\frac{a_{1}}{N}+𝒪\,\left(\frac{1}{N^{2}}\right)$$

and obtaining the unbiased term by a linear fit of the channel capacity calculated using increasing fraction of the data. By applying this procedure, we found a very small sample-size correction to channel capacity in all genetic backgrounds (Figure 1—figure supplement 4A–B). The same analysis applied to the redundancy/synergy (Figure 1—figure supplement 4C–D), showed that our conclusions are independent on the sample size. All our linear fits of channel capacity and redundancy/synergy (Figure 1—figure supplement 4E) from 60% to 100% of the data were above the 95% of confidence level, indicating that our data is far from the undersampled regime. Covariance sensitivity analysis To explore how channel capacity and redundancy depend on linear correlation and noise among ADF, ASI and NSM neurons requires a way to scale these two properties in silico from the baselines obtained in each genotype in experimental measurements. To do so, we first approximated the gene expression densities as multivariate normal distributions. This approximation captures most of the global features of our three-dimensional responses and allows us to control noise and correlations in terms of covariance matrices. The Gaussian assumption was also used in our previous decoding analysis (Entchev et al., 2015). The agreement between our present study and the decoding analysis shows indirectly that the Gaussian approximation can be used here for information-theoretic purposes. We used the maximum-likelihood estimates of the covariance matrices C for each genotype as a reference and then we transformed each entry of the covariance matrix according to the rule

(27) $$C_{i\,j}\to \left[\alpha \,\delta_{i\,j}+\alpha \,\beta \,\left(1-\delta_{i\,j}\right)\right]\,C_{i\,j}.$$

The transformation above rescales all the standard deviations of the responses by a factor α and the Pearson’s correlation index for all pairs of neurons by a factor β. Thus we studied the sensitivity of information-theoretic variables to noise and correlation by varying α and β over a biologically relevant range. In the main text we presented the sensitivity analysis of redundancy, in Figure 3A and Figure 3—figure supplement 1A–C we show the color-coded contour maps of channel capacity, signal and noise correlation obtained by varying the parameters α and β from 0.5 to 2 independently. We checked numerically the positivity of the covariance matrix for all pairs of α and β. The major factor that controls information capacity in wild-type is noise, which provides a rationale for the noise regulation by *daf-7* revealed in our previous study (Entchev et al., 2015). Scaling the linear correlation has a more pronounced effect in all the mutants and especially in the *daf-7(-)* mutant. This is due to the synergistic encoding in *daf-7(-)* mutants - since interactions between neurons are a crucial to a synergistic strategy, the system becomes much more sensitive to linear correlations. This effect is particularly evident in *daf-7(-)* mutants, where the signal correlation is almost unchanged under noise rescaling (Figure 3—figure supplement 1C), making noise correlation the more prominent contributing factor. Gaussian model We can explore the consequences of redundant or synergistic strategies by modeling how the information encoded by ADF, ASI and NSM neuron is read by an ideal output which conveys the information from the sensory neurons. The essential features of the ADF-ASI-NSM system are captured by using the model depicted in Figure 4—figure supplement 1A–B. Here, the information about a binary input B is encoded by three sensors $S_{1}$, $S_{2}$ and $S_{3}$ whose joint response is a multivariate normal distribution with mean vector $\mu_{b}$

(28) $$\mu_{b}=\left\{ \begin{array}{cc} \left\{a,a,a\right\} & if\;b=1\\ \left\{-a,-a,-a\right\} & if\;b=0 \end{array}\right.$$

where the parameter a is associated with the dynamic range of the response. The covariance matrix $C_{i\,j}\equiv \mathrm{cov}\,\left(S_{i},S_{j}\right)$ associated to the joint distribution is assumed to be stimulus-independent. This simplification is consistent with the observation that variances and correlations between neurons do not change considerably across food levels. The covariance matrix was parametrized as

(29) $$cov(S_{i},S_{j})=\left(\begin{array}{ccc} \sigma_{1}^{2} & \rho_{12}\sigma_{1}\sigma_{2} & \rho_{13}\sigma_{1}\sigma_{3}\\ \rho_{12}\sigma_{1}\sigma_{2} & \sigma_{2}^{2} & \rho_{23}\sigma_{2}\sigma_{3}\\ \rho_{13}\sigma_{1}\sigma_{3} & \rho_{23}\sigma_{2}\sigma_{3} & \sigma_{3}^{3} \end{array}\right),$$

where $\rho_{i\,j}$ are the correlation coefficients between $S_{i}$ and $S_{j}$ such that det⁡C>0. The information about the binary input encoded by the three Gaussian sensors is then integrated linearly by the output variable

(30) $$\mathrm{output}=\alpha \,S_{1}+\beta \,S_{2}+\gamma \,S_{3}$$

where α,β,γ>0 and α+β+γ=1. This choice implies that the output is also normally distributed with mean ±a dependently on the value of the input, whereas its variance reads

var(output)=α2var(S1)+β2var(S2)+γ2var(S3)+(31)+2αβcov(S1,S2)+2βγcov(S2,S3)+2αγcov(S1,S3)

In our setting we assume the two states of the input b∈{0,1} to be equally probable, leading to an information entropy of 1 bit. The information encoded by the sensors about the input, $I=M\,I\,\left(S_{1},S_{2},S_{3};B\right)$, is upper bounded by the input entropy, moreover the input information encoded by each component, $I_{k}=M\,I\,\left(S_{k};B\right)$, is always smaller than the joint information. We can combine these constraints into the inequality

(32) $$\max_{k}(I_{k})\leq I\leq 1\;bit.$$

Furthermore, since the output is a function of the sensor responses, the mutual information between input and output $I_{o}=M\,I\,\left(\mathrm{output};B\right)$ is bounded by the information encoded by the three sensors

(33) $$I_{o\,u\,t\,p\,u\,t}\leq I.$$

In order to understand the consequences of synergy and redundancy from the perspective of the output node in the network which receives the input information from the three sensors, we explored the parametric space of the model and calculated the information encoded by the sensors and by the output. By using the variance of the first sensor as a reference scale we set $\sigma_{1}=1$ and sampled the eight parameters left uniformly within the range

(34)0.1 < σ2<1(35)0.1 < σ3 < σ2(36)−0.7 < ρij < 0.7(37)0 < α < 1(38)0 < β < 1−α(39)0 < a < 2σ3,

The range above was selected based on the following considerations:

1. All variances have a lower bound (set to 0.1) to avoid singular regimes where the 3D normal distribution becomes too narrow around the mean.
2. From Equations (34,35) $\sigma_{1}\;>\sigma_{2}\;>\;\sigma_{3}$, which implies $I_{1}\;<\;I_{2}\;<\;I_{3}$.
3. The upper bound of 0.7 on the absolute correlation coefficients $\rho_{i\,j}$ was used to keep the correlations within a biologically relevant range. Correlations between ADF, ASI and NSM are lower than 0.5 in all food conditions and genetic backgrounds.
4. The conditions in Equations (34–36) do not guarantee the covariance matrix in Equation (29) to be positive definite, therefore in our sampling algorithm we rejected all parameter sets with det⁢(cov)<0.
5. In our sampling we choose the value of a to be lower than $2\,\sigma_{3}$. Larger values of a generate extreme regimes where $I_{3}$ is approximately one bit, and the inequality for the joint information $I_{3}\leq I\leq 1$ implies a positive redundancy

(40)
$$R=I_{1}+I_{2}+I_{3}-I\approx I_{1}+I_{2}\;>\;0.$$

In this condition the output information is very sensitive to the value of γ=1-α-β. For γ≈1, the output is only receiving input from $S_{3}$, leading to an efficient transmission of 1 bit of information. Lower values of γ lead to a decrease of the transmitted information due to the noisier contribution of $S_{1}$ and $S_{2}$ to the output. In Figure 4 and Figure 4—figure supplement 1 we show the calculation of information-theoretic quantities from a sample of ∼500000 parametric sets. Red and blue populations correspond respectively to redundant and synergistic configurations. The majority of the sampled configurations (65%) displays a positive redundancy. As discussed in the main text, the minimum value of the output information increases proportionally to the level of redundancy. This feature matches the intuitive view that redundant system allow to transmit infomation more reliably. The redundancy value is lower bounded by the negative of the total information encoded by the sensors and upper bounded by two bits (Figure 4—figure supplement 1C), due to the inequalities

(41) $$R=I_{1}+I_{2}+I_{3}-I\;<I_{1}\;+\;I_{2}\;<2.$$

Synergistic regimes occupy the region of low signal correlation and positive noise correlation (Figure 4—figure supplement 1D–F) and are generally characterized by low values of the information carried by single sensors (Figure 4—figure supplement 1E). In Figure 4—figure supplement 1G, we show the distribution of redundant/synergistic regimes with respect to the parameters a and $\sigma_{3}$, which represent dynamic range and noise in the model. The ratio between these two parameters quantifies the signal-to-noise ratio of the system

(42) $$S\,N\,R\equiv \frac{a}{\sigma_{3}}.$$

In the absence of extra constraints, redundant configurations are permitted for any value of the signal-to-noise ratio, whereas the population of synergistic regimes is depleted for high values of S⁢N⁢R (Figure 4—figure supplement 1H). When we require a non-zero lower bound to the information encoded by the sensors, $I\;>\;I_{min}$, we see the appearance of a critical value for $S\,N\,R=s^{*}\,\left(I_{0}\right)$ which separates two regions (see Figure 4—figure supplement 1H, right panel): a synergy-dominated region, for $SNR\;<\;s^{*}(I_{0})$, and a mixed region where both coding strategies are permitted $SNR\;>\;s^{*}(I_{0})$. The critical S⁢N⁢R value depends on threshold applied to the sensor information, in particular, $s^{*}\,\left(I_{0}\right)$ increases for increasing threshold $I_{0}$. This observation can be used to predict how changes in signal-to-noise ratio affect coding strategy. Consider a system operating redundantly at high S⁢N⁢R. Our model shows that independently of the details of the system, if we apply a perturbation to the system which reduces the S⁢N⁢R below $s^{*}\,\left(I_{0}\right)$, then in order to carry at least $I_{0}$ bits of information the system will necessarily adopt a synergistic strategy. Remarkably, this feature of the model is in perfect agreement with the switch from redundancy to synergy observed in the *daf-7(-)* mutant with respect to the wild-type animal. Reduction of S⁢N⁢R accompanied by a sufficient level of information encoded is always associated to a switch to synergy. This behaviour is easy to explain. When $a\ll \sigma_{3}$ the most informative sensor $S_{3}$ stores a very small amount of information due to the small S⁢N⁢R however the joint information can still reach one bit by increasing the eccentricity of the distributions, i.e. by increasing the linear correlations between sensors. This has the clear consequence of increasing the noise correlation, therefore shifting redundancy to negative values.
