# Supplementary material for: Genetic control of encoding strategy in a food-sensing neural circuit
Source: eLife. 2017 Feb 7;6:e24040. doi: 10.7554/eLife.24040 (PMC5295820; doi:10.7554/eLife.24040)
Supplement: Related texts

# Article commentary: Decision letter

**DOI:** 10.7554/eLife.24040.020

**Affiliations:** Howard Hughes Medical Institute, Columbia University, United States

In the interests of transparency, eLife includes the editorial decision letter and accompanying author responses. A lightly edited version of the letter sent to the authors after peer review is shown, indicating the most substantive concerns; minor comments are not usually included.

[Editors’ note: minor issues and corrections have not been included, so there is not an accompanying Author response.]

Thank you for submitting your article "Genetic Control of Encoding Strategy in a Food-sensing Neural Circuit" for consideration as a Research Advance by *eLife*. Your article has been reviewed by three peer reviewers, and the evaluation has been overseen by a Reviewing Editor (Oliver Hobert) and a Senior Editor. The reviewers have opted to remain anonymous.

The reviewers have discussed the reviews with one another and are in agreement that this work qualifies as an interesting Research Advance to your original *eLife* paper.

The only comment we would like you address is extremely minor in nature: Figure 1—figure supplement 3: panels A-D are shown, but the legend refers to A-E.
